# Influence of *trans‐*ferulic acid on the quality of ram semen upon cold preservation

**DOI:** 10.1002/vms3.1117

**Published:** 2023-03-13

**Authors:** Ali Salimi, Mohsen Eslami, Farhad Farrokhi‐Ardabili

**Affiliations:** ^1^ Department of Theriogenology, Faculty of Veterinary Medicine Urmia University Urmia Iran; ^2^ Department of Animal Sciences, Faculty of Agriculture Urmia University Urmia Iran

**Keywords:** cold preservation, ram, spermatozoa, trans‐ferulic acid

## Abstract

**Background:**

Due to lower antioxidant capacity and higher amounts of polyunsaturated fatty acids, ram spermatozoa are very susceptible during cooling process.

**Objectives:**

The objective was to examine the effect of the trans‐ferulic acid (*t*‐FA) on the ram semen during liquid preservation.

**Methods:**

Semen samples were collected from the Qezel rams, pooled, and extended with the Tris‐based diluent. Pooled samples enriched with different amounts of the *t*‐FA (0, 2.5, 5, 10, and 25 mM) and preserved at 4°C for 72 h. Spermatozoa's kinematics, membrane functionality, and viability were assessed by CASA system, hypoosmotic swelling test, and eosin‐nigrosin staining, respectively. Moreover, biochemical parameters were measured at 0, 24, 48, and 72 h.

**Results:**

Results showed that 5 and 10 mM *t*‐FA improved forward progressive motility (FPM) and curvilinear velocity compared to the other groups at 72 h (*p* < 0.05). Samples treated with 25 mM *t*‐FA showed the lowest total motility, FPM, and viability at 24, 48, and 72 h of storage (*p* < 0.05). Higher total antioxidant activity levels were observed in the 10 mM *t*‐FA‐treated group compared to the negative control at 72 h (*p* < 0.05). Treatment with 25 mM *t*‐FA increased malondialdehyde amounts and decreased superoxide dismutase activity compared to other groups at the final time assessment (*p* < 0.05). Nitrate‐nitrite and lipid hydroperoxides values were not affected by treatment.

**Conclusions:**

The current study indicates the positive and negative influences of different concentrations of *t*‐FA on the ram semen upon cold storage.

## Graphical abstract

Different concentrations of *trans*‐ferulic acid (*t*‐FA) showed diverse effects on chilled ram semen.

Enrichment with 5 or 10 mM t‐FA improved kinematics of spermatozoa and TAC variable.

Administration of 25 mM t‐FA reduced ram semen quality during cold preservation.

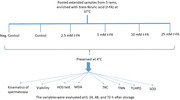

## 1 INTRODUCTION

Artificial insemination (AI) was introduced and performed in the sheep industry to achieve maximum genetic efficiency using semen samples of the elite rams. Furthermore performing AI reduces the chance of reproductive diseases (Maxwell & Watson, 1996). In some circumstances, and in order to prevent the detrimental effects of the freezing/thawing, the insemination could be done with a diluted fresh or cold semen, instead of the frozen one (King et al., 2004). It has been revealed that the induced injuries to spermatozoa preserved at 4°C are not comparable to frozen‐thawed samples (Aisen et al., 2002; Gil et al., 2003). However, the decrease in motility and fertility of spermatozoa during cold preservation would be an unfavourable and limiting factor in the development of AI (Maxwell & Salamon, 1993; Salamon & Maxwell, 2000). Unfortunately, generation of different active oxygen species during cooling and/or freezing‐thawing conduces lipid peroxidation of spermatozoa membrane (Alvarez & Storey, 1995). Lipid peroxidation (LP) affects the spermatozoa by inducing irreversible loss of kinematics, damaging the genome and finally reducing its fertility (Maxwell & Salamon, 1993). The existence of remarkable amounts of polyunsaturated fatty acids (PUFAs) in the ram spermatozoa membrane, concomitant with the low antioxidant capacity of semen, makes it sensitive to the destructive effects of the LP during storage. Furthermore, a negative relationship between the malondialdehyde (MDA) levels, and the quality of the fresh or cold/frozen semen samples have been reported (Lewis et al., 1995; Bucak et al., 2010). Therefore, an exogenous effective antioxidant system is mandatory to prevent or reduce the severity of LP and increase the spermatozoa function, to maintain its fertility (in vitro) at the optimum level (Alvarez & Storey, 1995; Holt, 2000; Shayan‐Nasr et al., 2021). In this regard, enrichment of the semen extender with the aim of the improving antioxidative levels has been developed during cold‐liquid or frozen storage of semen samples (Benhenia et al., 2018; Câmara et al., 2011; Eslami et al., 2016; Ghaniei et al., 2019). Controversial findings were reported following semen supplementation with exogenous antioxidants. In this subject, previous reports revealed that enrichment with exogenous compounds might improve (Jang et al., 2020; Mohammadi & Soltani, 2021), did not affect (Moradi et al., 2022), or even deteriorated semen variables during preservation (Alamaary et al., 2020; Hermansson et al., 2021). Therefore, increasing knowledge about the effects of exogenous antioxidants on the quality of stored semen would help us to choose the appropriate compounds with optimum doses.

Ferulic acid (FA; 4‐hydroxy‐3‐methoxynamide acid) is a natural compound whose precursor is the plant cell wall (Hlasiwetz & Barth, 1866). There is a natural phenolic compound with free and conjugated forms in the plants. Extensive physiological activities of FA such as antioxidants (direct or indirect), anti‐allergy, anti‐cancer, liver protector, antimicrobial, metal chelating, and signal transduction at the cell level and effect on the expression of genes were indicated (Kumar & Pruthi, 2014). Furthermore, *trans*‐ferulic acid (*t*‐FA) has been reported to improve the protection of mitochondria, acrosomes, and the plasma membranes functionality of rooster and stallions’ spermatozoa against deleterious effects of the free radicals upon cold storage (Affonso et al., 2017; Shayan‐Nasr et al., 2021). Another study revealed that *t*‐FA could alleviate cold‐induced oxidative stress in the liver and kidney of mice by increasing enzymatic antioxidants (Xue et al., 2018). Moreover, the capability of *t*‐FA in reducing the amounts of LP and upregulation of the enzymatic antioxidants has been revealed (Roy et al., 2014; Sgarbossa et al., 2015). However, literature review revealed that effect of *t*‐FA has not been investigated on the ram semen throughout in vivo and in vitro experiments. The objective was to evaluate whether different amounts of *t*‐FA would increase the kinematics of ram spermatozoa and modulate the biochemical variables of the sample upon cold preservation.

## 2 MATERIALS AND METHODS

### 2.1 Collection of semen from the rams

Five rams of the Qezel breed (2–3 years old) were used to collect the semen. Ejaculates were collected from the rams by using an artificial vagina in the presence of oestrus ewes (Paulenz et al., 2002), for two times a week during the breeding seasons (autumn and winter). After collection, samples were taken to the lab and incubated at 37°C in a water bath. The volume, number of spermatozoa, and mass motility of samples were assessed after collection. Semen volume was read using calibrated conical tubes; then, a drop of raw sample was placed on the slide (without the cover slide) and observed under a microscope (lens 10; Labomed, Labomed, Inc., Culver City, CA, USA) to estimate the mass motility. Spermatozoa's concentration was determined using a haemocytometer. Samples with a range of 0.75−2 mL in volume, mass motility greater than 3 (range of 1–5), and the concentration greater than 2.5 × 10^9^ spermatozoa/mL were mixed and used for the experiment.

### 2.2 Extension of semen and grouping of experiment

All the chemicals used in this study were purchased from the Merck or Sigma‐Aldrich Companies. The Tris‐based extender was prepared by dissolving Tris (hydroxymethyl aminomethane), citric acid, fructose, penicillin, and streptomycin in 86 mL of double distilled water (3.63, 1.99, 0.5, 100,000 IU, and 100 mg, respectively). Then, 14 mL of egg yolk was added to the buffer (Evans & Maxwell, 1987). Pooled semen samples were diluted with extender to set the concentration spermatozoa at 500 million/mL. The negative control group did not received any treatment, while the control group received dimethyl sulfoxide (DMSO; the solvent of the t‐FA). The other experimental groups received 2.5, 5, 10, and 25 mM DMSO dissolved *t*‐FA (Sigma‐Aldrich, 128708, CAS NO: 537‐98‐4, Germany). The different stock solution of DMSO dissolved *t*‐FA was prepared (0, 0.5, 1, 2, and 5 M), and all samples received equal amounts of DMSO (5 μL) with varying concentration of *t*‐FA (0, 2.5, 5, 10, and 25 mM). The treated samples were preserved in refrigerator for 3 days. Kinematics of spermatozoa (evaluated by CASA), percentage of live spermatozoa (by eosin‐nigrosin staining; Salamon & Maxwell, 2000), and plasma membrane functionality (by hypo‐osmotic swelling test [HOST]) were assessed at 0 h and repeated every 24 h. Furthermore, the levels of thiobarbituric reactive substances (MDA), total nitrate‐nitrite (TNN; as an indicator of nitrosative stress), total antioxidant capacity (TAC), total lipid hydroperoxide (TLHPO), and activity of superoxide dismutase enzyme (SOD) were assessed in the homogenate of spermatozoa‐diluent of the treated samples at 0, 24, 48, and 72 h.

### 2.3 Kinematics of spermatozoa

A computer‐assisted sperm analyzer (CASA; Test Sperm 3.2, Videotest, St. Petersburg, Russia) was utilized to evaluate the kinematics. The system includes a microscope (phase contrast, Labomed, Labomed, Inc.) equipped with a stage warmer, and a digital camera (M‐Shot, MD50‐T, China). The motility was captured at a rate of 50 frames/s by the system. Diluted sample (10 μL) was put on a warmed slide (37°C) and covered with a cover slip (22 × 22 mm), and the motility characteristics were assessed at 200×. The CASA variables included total motility (TM), forward progressive motility (FPM), curvilinear velocity (VCL, μm/s), average path velocity (VAP, μm/s), straight‐line velocity (VSL, μm/s), straightness (STR = [VSL/VAP] × 100, %), and linearity (LIN = [VSL/VCL] × 100, %).

### 2.4 Viability of spermatozoa

To assess the percentage of viability, samples were stained with eosin‐nigrosin staining (Evans & Maxwell, 1987). For this, 25 μL of diluted sample was mixed with 50 μL of eosin‐nigrosin stain, and the smear was prepared immediately and dried. The viability (%) was calculated by counting a total number of at least 200 spermatozoa under a light microscope. Spermatozoa without colour were regarded as live, and the others (coloured spermatozoa) were regarded as dead.

### 2.5 Membrane functionality of spermatozoa

The HOST solution was prepared according to the methods of Correa and Zavos (1994). One part of the diluted sample was mixed with the 10 parts of the HOST solution at 37°C and preserved in a water bath for 1 h. The number of coiled‐tailed spermatozoa in a total of 200 spermatozoa (counted by a phase contrast microscope) was used to calculate the percent of intact plasma membrane.

### 2.6 Preparation of sample for assessment of biochemical variables

After evaluation of the kinematics, viability, and membrane functionality of spermatozoa, the remainder of the treated samples were homogenized with an electronic homogenizer (T10 Basic, IKA®, Werke GmbH & Co. KG, Staufen, Germany) at 5000 *g* for 4 min. The prepared homogenates were stored at −20°C until the measurement of biochemical variables.

### 2.7 Amounts of MDA in the homogenate

MDA as an oxidant indicator was assessed by the thiobarbituric acid reactive substances (Stern et al., 2010). In brief, the thiobarbituric reagent (two parts) was mixed with the sample (one part). The reaction was completed by keeping the test tubes in a boiling water bath (100°C) for 15 min. In the next step, centrifugation was done at 2000 *g* for 10 min, and the supernatant was collected in a new tube. Finally, the optical density (OD) of supernatant was recorded using a spectrophotometer (at 532 nm wavelength; Pharmacia, Pharmacia LKB, NOVASPEC II) against blank sample. The amounts of MDA were calculated (Concentration = OD/1.56 × 10^5^ M) and expressed as μmol/L.

### 2.8 TAC levels in the homogenate of samples

Amounts of TAC were determined using the previously published method (Koracevic et al., 2001). In brief,490 μL of PBS solution was added to 10 μL of the sample. Additionally, sodium benzoate (0.5 mL), acetic acid (1 mL), Fe‐EDTA (0.2 mL), and H_2_O_2_ (0.2 mL) were added to the tubes, respectively. Uric acid solution (1 mmol/L) was used as the positive control according to the protocol. The tubes were incubated in a water bath at 37°C for 60 min. Acetic acid and thiobarbituric prepared solutions were subsequently added to the tubes. The tubes were incubated in a boiling water bath (100°C) for 10 min. After completion of reaction, the density of the samples was recorded using a spectrophotometer (532 nm wavelength; Pharmacia, Pharmacia LKB, NOVASPEC II). The TAC values were calculated according to the following formula and presented as mmol/L: TAC (mmol/L) = (CUA) × (*K* − *A*)/(*K* − UA). The *K* = absorbance of negative control (*K*1 − *K*0; without sample), *A* = absorbance of sample (*A*1 − *A*0), UA = absorbance of uric acid solution (UA1 − UA0), CUA = concentration of uric acid (mmol/L).

### 2.9 Measurement of TNN

Amounts of TNN, as a nitrosative stress index in the samples, were assessed using Griess reaction (Green et al., 1982). Fresh Griess reagent was prepared by mixing equal volume of 1% sulfanilamide dissolved in 5% H_3_PO_4_ with 0.1% naphthylethylenediamine dihydrochloride dissolved in distilled water. Equal amounts of sample and Griess reagent were mixed in a plate, incubated at the laboratory temperature for 10 min under dark conditions. The OD of the generated solution was recorded at 540 nm. The amounts of TNN in the samples were calculated according to the drawn standard curve. The TNN values were shown as μmol/L.

### 2.10 Measurement of SOD activity

The SOD activities were assessed by the pyrogallol oxidation method (Marklund & Marklund, 1974). In brief, tris buffer was prepared by dissolving appropriate amounts of ethylene diamine ethylenediaminetetraacetic acid and tris; then, the PH of buffer was adjusted at 8.5. According to the protocol after adding of sample to buffer, the OD was set at zero. In the following, pyrogallol solution was added, and the OD was read (420 nm) using spectrophotometer (Pharmacia, Pharmacia LKB, NOVASPEC II) at 1.5 and 3.5 min after initiation of the reaction. By the standard formula according to the reference, the activity of SOD was calculated and showed as unit/mL. One unit of SOD was calculated as the amounts of enzyme that causes 50% inhibition of the pyrogallol auto‐oxidation rate.

### 2.11 Amounts of total lipid hydroperoxide in the samples

Amounts of TLHPO were measured using Fox reagent (Nourooz‐Zadeh et al., 1995). One part of the sample was mixed with nine parts of reagent, incubated for 30 min in darkness at laboratory temperature, and the absorbance was recorded at 560 nm. The Fox reagent contained 90% (v/v) methanol, 4 mM butylated hydroxytoluene, 25 mM sulfuric acid, 250 mM ammonium ferrous sulphate hexahydrate (Fe(NH4)2(SO4)2), 100 mM xylenol orange. The standard plot curve was drawn using hydrogen peroxide. The concentrations of TLHPO were depicted as μmol/L.

### 2.12 Statistical analysis

Variables related to the spermatozoa itself (kinematics, viability, membrane integrity), as well as homogenate (TAC, MDA, TLHPO, TNN, SOD), were shown as the least square mean ± SEM. Means were analyzed using a two‐way analysis of variance. The Tukey's test was used as a post hoc test to determine the effect of treatment and time in all the recorded parameters among groups. The SigmaStat software (Version 3.5; Chicago, IL, USA) was used to analyze the data. The Kolmogorov–Smirnov (with Lilliefors’ correction) and Levene median tests were used to evaluate data for normality and equal variance assumption of the estimated underlying population within the SigmaStat, respectively. When the normality tests of the raw data failed, the statistical test was conducted on ranks. A *p*‐value less than 0.05 was considered to be significant.

## 3 RESULTS

Results showed that exposure of semen to the 25 mM *t*‐FA decreased TM, FPM, VAP, VSL, and VCL compared to other groups at 24, 48, and 72 h (*p* < 0.05; Tables 1 and 2). Semen enrichment with 5 and 10 mM *t*‐FA increased FPM, and VCL compared to control and negative control groups at the last time (*p* < 0.05; Tables 1 and 2). The STR variable was more significant in the 10 mM *t*‐FA group compared to the 25 mM *t*‐FA‐treated group at the last time (*p* < 0.05; Table 2). Linearity was not influenced by the treatment among groups (*p* > 0.05; Table 2). Results indicated that TM, FPM, VCL, VSL, and VAP were reduced at 48, and 74 h compared to the first time in all experimental groups (*p* < 0.05; Tables 1 and 2).

**TABLE 1:** Percentage of total and forward progressive motility, viability, and intact membrane of ram spermatozoa supplemented with different concentrations of trans‐ferulic acid (TFA) before preservation at 4°C.

		Time of storage (h)				
Parameter	Treatment	0	24	48	72	S.E. of LS means
Total motility (%)	Neg. control	91.35 ^Aa^	80.65 ^Ab^	71.14 ^Ac^	68.94 ^Ac^	2.15
	Control (DMSO)	89.68 ^Aa^	80.91 ^Ab^	74.08 ^Abc^	69.42 ^Ac^	2.15
	TFA 2.5 mM	88.93 ^Aa^	78.68 ^Ab^	74.56 ^Abc^	68.38 ^Ac^	2.15
	TFA 5 mM	88.87 ^Aa^	79.22 ^Ab^	77.15 ^Ab^	70.01 ^Ac^	2.15
	TFA 10 mM	90.89 ^Aa^	85.34 ^Aa^	77.54 ^Ab^	74.55 ^Ab^	2.15
	TFA 25 mM	87.22 ^Aa^	63.42 ^Bb^	55.72 ^Bc^	43.73 ^Bd^	2.15
Forward progressive motility (%)	Neg. control	75.32 ^Aa^	53.96 ^Ab^	43.43 ^Ac^	39.36 ^Bc^	1.96
	Control (DMSO)	76.55 ^Aa^	51.92 ^Ab^	43.54 ^Ac^	38.74 ^Bc^	1.96
	TFA 2.5 mM	76.19 ^Aa^	50.53 ^Ab^	42.49 ^Ac^	38.44 ^Bc^	1.96
	TFA 5 mM	78.30 ^Aa^	52.03 ^Ab^	49.74 ^Abc^	46.92 ^Ac^	1.96
	TFA 10 mM	74.01 ^Aa^	54.56 ^Ab^	49.84 ^Abc^	47.46 ^Ac^	1.96
	TFA 25 mM	76.93 ^Aa^	38.81 ^Bb^	24.83 ^Bc^	15.97 ^Cd^	1.96
Viability (%)	Neg. control	94.79 ^Aa^	83.81 ^Ab^	75.01 ^Ac^	71.10 ^Ac^	1.73
	Control (DMSO)	94.75 ^Aa^	84.12 ^Ab^	77.75 ^Ac^	74.88 ^Ac^	1.73
	TFA 2.5 mM	92.15 ^Aa^	82.33 ^Ab^	79.59 ^Abc^	74.22 ^Ac^	1.73
	TFA 5 mM	93.55 ^Aa^	81.96 ^Ab^	79.14 ^Ab^	76.50 ^Ab^	1.73
	TFA 10 mM	93.16 ^Aa^	86.06 ^Ab^	79.86 ^Ac^	76.39 ^Ac^	1.73
	TFA 25 mM	92.75 ^Aa^	72.91 ^Bb^	64.76 ^Bc^	58.45 ^Bd^	1.73
HOS (%)	Neg. control	90.73 ^Aa^	83.07 ^Ab^	77.80 ^Ab^	70.31 ^Ac^	2.04
	Control (DMSO)	89.03 ^Aa^	85.72 ^Aa^	78.29 ^Ab^	73.74 ^Ab^	2.04
	TFA 2.5 mM	90.64 ^Aa^	83.35 ^Ab^	75.42 ^Ac^	73.85 ^Ac^	2.04
	TFA 5 mM	91.13 ^Aa^	82.82 ^Ab^	79.05 ^Ab^	77.34 ^Ab^	2.04
	TFA 10 mM	89.65 ^Aa^	84.61 ^Aab^	77.84 ^Ab^	77.52 ^Ab^	2.04
	TFA 25 mM	88.60 ^Aa^	78.33 ^Ab^	70.90 ^Ac^	57.41 ^Bd^	2.04

*Note*: Values with different uppercase superscripts indicate a difference (*p* < 0.05) among groups at each time point for each variable. Values with different lowercase superscripts indicate a significant difference (*p* < 0.05) between the data at the same raw for each variable.

Abbreviations: DMSO, dimethyl sulfoxide; Neg., negative; S.E. of LS means, standard error of the least squares means.

**TABLE 2:** Kinematics of ram spermatozoa (analyzed by CASA) supplemented with different concentrations of trans‐ferulic acid (TFA) before preservation at 4°C.

			Time of storage (h)			
Parameter	Treatment	0	24	48	72	S.E. of LS means
VAP (μm/s)	Neg. control	32.33 ^Aa^	29.77 ^Aa^	21.33 ^Ab^	20.99 ^Ab^	1.28
	Control (DMSO)	31.52 ^Aa^	28.54 ^Aa^	22.61 ^Ab^	20.98 ^Ab^	1.28
	TFA 2.5 mM	32.84 ^Aa^	28.01 ^Ab^	22.65 ^Abc^	19.58 ^Ac^	1.28
	TFA 5 mM	32.30 ^Aa^	27.11 ^Ab^	23.73 ^Abc^	21.50 ^Ac^	1.28
	TFA 10 mM	33.78 ^Aa^	31.45 ^Aa^	25.78 ^Ab^	22.52 ^Ab^	1.28
	TFA 25 mM	30.82 ^Aa^	21.43 ^Bb^	10.00 ^Bc^	6.87 ^Bc^	1.28
VSL (μm/s)	Neg. control	22.59 ^Aa^	20.11 ^Aab^	17.94 ^Bb^	16.76 ^Bb^	0.89
	Control (DMSO)	24.71 ^Aa^	20.41 ^Ab^	17.31 ^Bb^	16.54 ^Bb^	0.89
	TFA 2.5 mM	23.49 ^Aa^	20.30 ^Aab^	18.18 ^Bb^	16.83 ^Bb^	0.89
	TFA 5 mM	23.46 ^Aa^	20.54 ^Ab^	18.77 ^ABb^	18.41^ABb^	0.89
	TFA 10 mM	24.23 ^Aa^	23.55 ^Aa^	21.90 ^Aa^	20.61^Ab^	0.89
	TFA 25 mM	22.85 ^Aa^	9.70 ^Bb^	7.32 ^Cbc^	5.07 ^Cc^	0.89
VCL (μm/s)	Neg. control	106.43 ^Aa^	79.73 ^Ab^	63.20 ^Ac^	50.39 ^Bd^	3.16
	Control (DMSO)	109.55 ^Aa^	76.71 ^Ab^	64.31 ^Ac^	52.23 ^Bd^	3.16
	TFA 2.5 mM	103.89 ^Aa^	78.51 ^Ab^	62.87 ^Ac^	49.50 ^ABd^	3.16
	TFA 5 mM	107.96 ^Aa^	76.07 ^Ab^	68.82 ^Abc^	63.71 ^Ac^	3.16
	TFA 10 mM	100.77 ^Aa^	83.48 ^Ab^	67.71 ^Ac^	60.11 ^Ac^	3.16
	TFA 25 mM	86.42 ^Ba^	56.99 ^Bb^	42.80 ^Bc^	32.49 ^Cd^	3.16
STR (%)	Neg. control	72.61 ^Aa^	76.42 ^Aa^	70.75 ^Aa^	72.89 ^ABa^	2.09
	Control (DMSO)	74.83 ^Aa^	75.39 ^Aa^	73.12 ^Aa^	71.31 ^ABa^	2.09
	TFA 2.5 mM	74.13 ^Aa^	75.43 ^Aa^	76.75 ^Aa^	73.24 ^ABa^	2.09
	TFA 5 mM	71.37 ^Aa^	76.90 ^Aa^	72.81 ^Aa^	70.96 ^ABa^	2.09
	TFA 10 mM	76.87 ^Aa^	78.51 ^Aa^	75.08 ^Aa^	78.43 ^Aa^	2.09
	TFA 25 mM	76.14 ^Aa^	73.55 ^Aa^	72.61 ^Aab^	65.14 ^Bb^	2.09
LIN (%)	Neg. control	24.91 ^Aa^	26.64 ^Aa^	22.54 ^Aa^	22.06 ^Aa^	1.33
	Control (DMSO)	25.33 ^Aa^	24.78 ^Aa^	21.87 ^Aa^	20.64 ^Aa^	1.33
	TFA 2.5 mM	25.37 ^Aa^	23.93 ^Aa^	24.01 ^Aa^	22.21 ^Aa^	1.33
	TFA 5 mM	24.09 ^Aa^	24.44 ^Aa^	22.45 ^Aa^	20.19 ^Aa^	1.33
	TFA 10 mM	27.04 ^Aa^	27.59 ^Aa^	25.26 ^Aa^	22.57 ^Aa^	1.33
	TFA 25 mM	27.07 ^Aa^	23.53 ^Ab^	19.6 ^Ac^	13.66 ^Bd^	1.33

*Note*: Values with different uppercase superscripts indicate a difference (*p* < 0.05) among groups at each time point for each variable. Values with different lowercase superscripts indicate a difference (*p* < 0.05) between the data at the same raw for each variable.

Abbreviations: DMSO, dimethyl sulfoxide; LIN, linearity; Neg., negative; S.E. of LS means, standard error of the least squares means; STR, straightness; VAP, average path velocity; VCL, curvilinear velocity; VSL, straight‐line velocity.

Viability was influenced by the administration of high amounts of *t*‐FA at 24, 48, and 72 h (*p* < 0.05; Table 1). Furthermore, viability was reduced at 24, 48, and 72 h compared to the first time in all groups (*p* < 0.05; Table 1).

Membrane integrity was not influenced by the treatment except in the 25 mM *t*‐FA group at 72 h (*p* < 0.05; Table 1). Results over time showed that a lower percent of membrane integrity was detected at 48, and 72 h compared to the first time in the experimental groups (*p* < 0.05; Table 1).

The lowest TAC amounts were recorded in the 25 mM *t*‐FA‐exposed group than in the other groups at all times except 0 h (*p* < 0.05; Table 3). Greater amounts of TAC were detected in the 10 mM *t*‐FA group than in the negative control group at the last time (*p* < 0.05; Table 3). Changes in TAC over time indicated the reduction of antioxidant capacity in the 25 mM‐treated group at the 24, 48, and 72 h compared to the first time of the experiment (*p* < 0.05; Table 3).

**TABLE 3:** Amounts of total antioxidant capacity (TAC), malondialdehyde (MDA), and superoxide dismutase (SOD) activity in ram semen samples supplemented with different concentrations of trans‐ferulic acid (TFA) before preservation at 4°C.

		Time of storage (h)				
Parameter	Treatment	0	24	48	72	S.E. of LS means
TAC (mmol/L)	Neg. control	4.04 ^Aa^	3.95 ^ABa^	3.64 ^Aa^	3.69 ^Ba^	0.171
	Control (DMSO)	4.45 ^Aa^	4.31 ^Aa^	3.97 ^Aba^	3.96 ^ABa^	0.171
	TFA 2.5 mM	4.38 ^Aa^	4.46 ^Aa^	4.33 ^Aa^	4.24 ^ABa^	0.171
	TFA 5 mM	4.27 ^Aa^	4.06 ^ABa^	4.23 ^Aa^	4.19 ^ABa^	0.171
	TFA 10 mM	4.48 ^Aa^	4.15 ^Aa^	4.20 ^Aa^	4.32 ^Aa^	0.171
	TFA 25 mM	4.57 ^Aa^	3.35 ^Bb^	3.10 ^Bb^	2.41 ^Cc^	0.171
MDA (μmol/L)	Neg. control	0.43 ^Aa^	0.50 ^ABab^	0.53 ^Ab^	0.56 ^Ab^	0.01
	Control (DMSO)	0.44 ^Aa^	0.48 ^ABab^	0.51 ^Aab^	0.55 ^Ab^	0.01
	TFA 2.5 mM	0.43 ^Aa^	0.45 ^Aa^	0.48 ^Aab^	0.53 ^Ab^	0.01
	TFA 5 mM	0.46 ^Aa^	0.48 ^ABa^	0.48^Aa^	0.49 ^Aa^	0.01
	TFA 10 mM	0.44 ^Aa^	0.49 ^ABab^	0.51 ^Aab^	0.53 ^Ab^	0.01
	TFA 25 mM	0.45 ^Aa^	0.55 ^Bb^	0.66 ^Bc^	0.72 ^Bd^	0.01
SOD (Unit/mL)	Neg. control	7.64 ^Aa^	7.03 ^Aa^	6.74 ^Aa^	6.10 ^Aa^	0.29
	Control (DMSO)	7.28 ^Aa^	7.16 ^Aa^	6.28 ^ABa^	6.31 ^Aa^	0.29
	TFA 2.5 mM	7.08 ^Aa^	7.05 ^Aa^	6.22 ^ABa^	6.26 ^Aa^	0.29
	TFA 5 mM	7.19 ^Aa^	7.69 ^Aa^	6.23 ^ABa^	6.34 ^Aa^	0.29
	TFA 10 mM	6.98 ^Aa^	7.39 ^Aa^	6.59 ^Aa^	6.42 ^Aa^	0.29
	TFA 25 mM	7.04 ^Aa^	6.34 ^Aab^	5.38 ^Bbc^	4.44 ^Bc^	0.29

*Note*: Values with different uppercase superscripts indicate a difference (*p* < 0.05) among groups at each time point for each variable. Values with different lowercase superscripts indicate a significant difference (*p* < 0.05) between the data at the same raw for each variable.

Abbreviations: DMSO, dimethyl sulfoxide; Neg., negative; S.E. of LS means, standard error of the least squares means; TFA, trans‐ferulic acid.

Higher amounts of MDA levels were recorded in the 25 mM *t*‐FA‐exposed group than in the other groups during the third and fourth times of the study (*p* < 0.05; Table 3). Changes over time showed that except for the 5 mM *t*‐FA group, MDA levels were more significant at the last time of the study compared to the 0 h in other treated groups (*p* < 0.05; Table 3). Exposure of ram spermatozoa to 25 mM *t*‐FA resulted in lower SOD activity compared to other experimental groups at 72 h (*p* < 0.05; Table 3).

Significant changes among experimental groups or among studied time points within a group in TNN and TLHPO amounts were not detected (*p* > 0.05; Table 4).

**TABLE 4:** Amounts of total nitrate‐nitrite (TNN) and total lipid hydroperoxide (TLHPO) in ram semen samples supplemented with different concentrations of trans‐ferulic acid (TFA) before preservation at 4°C.

		Time of storage (h)				
Parameter	Treatment	0	24	48	72	S.E. of LS means
TNN (μmol/L)	Neg. control	294.11	290.11	303.72	334.78	20.61
	Control (DMSO)	303.60	314.53	292.19	313.52	20.61
	TFA 2.5 mM	277.91	298.29	282.41	318.66	20.61
	TFA 5 mM	284.13	260.61	315.68	313.49	20.61
	TFA 10 mM	280.73	310.77	323.44	330.42	20.61
	TFA 25 mM	292.17	271.40	313.23	366.39	20.61
TLHPO (μmol/L)	Neg. control	10.98	11.16	11.47	11.44	1.36
	Control (DMSO)	10.26	10.10	10.72	13.18	1.36
	TFA 2.5 mM	10.73	9.82	11.44	12.19	1.36
	TFA 5 mM	10.38	11.35	13.21	12.75	1.36
	TFA 10 mM	10.13	11.24	10.84	12.02	1.36
	TFA 25 mM	10.77	12.01	12.21	13.75	1.36

*Note*: There was no significant difference among groups at each time point or between the time points within every experimental group.

Abbreviations: DMSO, dimethyl sulfoxide; Neg., negative; S.E. of LS means, standard error of the least squares means.

## 4 DISCUSSION

The purpose of the current study was to assess the potential protective role of supplementing with *t*‐FA on the variables of ram semen (CASA output, viability, membrane integrity, and the levels of TAC, MDA, TNN, TLHPO, and activity of SOD) during child storage up to 72 h.

A previous study reported that the capability of spermatozoa in fertilization was related to the kinematics variables (Aitken et al., 1984, 1983; Larsen et al., 2000; Hirano et al., 2001; Kumar & Singh, 2015). Results of the current study showed that supplementing with 5 and 10 mM *t*‐FA increased FPM, as well as VCL, compared to the control and negative control groups at 72 h, which probably could be correlated with the fertilization capability of spermatozoa. Furthermore, our results indicated that the viability and membrane functionality of spermatozoa were not superior in groups that received *t*‐FA compared to the control groups. However, in relation to viability, the capability of FA in increasing the ratio of Bcl‐2/Bax was shown (Cheng et al., 2016). Previous studies have shown that semen supplementation with antioxidant compounds leads to higher motile and viable of the liquid or frozen‐thawed spermatozoa in different animals and birds (goat (Bucak et al., 2010), bull (Kiernan et al., 2013; Takahashi et al., 2012), dog (Cassani et al., 2005), red deer (Anel‐López et al., 2012, 2015), rooster (Eslami et al., 2016; Rad et al., 2016) and turkey (Donoghue & Donoghue, 1997)). Furthermore, the effectiveness of different compounds and antioxidants on improving ram semen kinematics, viability, membrane functionality, and even fertility have been reported (Asadzadeh, et al., 2021; Bucak et al., 2008; Maia et al., 2010; Maxwell & Stojanov, 1996; Shahat et al., 2022; Zadeh Hashem et al., 2017). Varying roles and effects of *t*‐FA have been reported on living organisms. In this regard, the influence of *t*‐FA on reproductive organs of animals (Zheng & Zhang, 1997), and as a protectant against β‐cyfluthrin‐mediated toxicity during rooster semen chilled preservation (Shayan‐Nasr et al., 2021) has been documented. Moreover, the positive effects of FA in the restoration of spermatozoa kinematics and viability of diabetic rats were reported (Roy et al., 2014). The most protective effects of *t*‐FA on different parameters of rooster spermatozoa upon co‐administration with mild toxic doses of β‐cyfluthrin have been reported at 10 and 25 mM doses (Shayan‐Nasr et al., 2021). However, ram semen enrichment with 25 mM *t*‐FA (dissolved in DMSO) resulted in a decrease in kinematics, viability, and intact membrane of treated samples. The ingredients of the ram semen extender were different compared to the rooster's ingredients. The egg yolk or its plasma fraction is one of the main and inseparable components of ruminants’ extender (Mortazavi et al., 2020; Salamon & Maxwell, 2000), while the inclusion of egg yolk was not recommended to the extender of the rooster semen (Santiago‐Moreno et al., 2012). Moreover, the preventive and protective effects of 25 mM *t*‐FA in rooster extender were observed during co‐treatment with toxic doses of an oxidant (Shayan‐Nasr et al., 2021), while in the current experiment exogenous oxidant or toxic substances did not apply. Another difference was attributed to the studied species. The present experiment was designed on ram semen (with very sensitive spermatozoa, due to the remarkable quantities of PUFAs, and the low amounts of cholesterol within its membrane), while the previous study was done on the rooster spermatozoa with relatively high resistance to cold shock injuries compared to the ram spermatozoa.

A report revealed that the cooling process during semen preservation at 4°C stimulates the generation of reactive oxygen species (ROS; Sicherle et al., 2011). Low amounts of ROS and reactive nitrogen species (RNS) are mandatory in the process of hyper‐activation, capacitation, and acrosome reaction of spermatozoa (Aziz et al., 2010; De Iuliis et al., 2009). However, their high production caused inappropriate environment and deteriorated the spermatozoa normal functions and impaired its viability, which is known as oxidative toxicity (Du Plessis et al., 2010). Reduction of spermatozoa motility and membrane functionality, disturbance of mitochondrial function, increase of MDA levels and lipid peroxidation, and damage to the acrosome membrane and DNA are the well‐known consequences of oxidative toxicity (OT) to spermatozoa (Aitken et al., 1993; Baumber et al., 2000; Griveau et al., 1995; Burnaugh et al., 2007; Balao da Silva et al., 2011; Marques et al., 2002). The higher sensitivity of ram and goat spermatozoa to OT upon cold/frozen preservation is attributed to the existence of remarkable amounts of PUFAs in the membrane of the spermatozoa (Gandini et al., 2000). Accordingly, supplementation with antioxidative compounds seems vital in order to alleviate the detrimental effects of OT upon semen storage. Ferulic acid is a derivative of hydrocinimic acids that are found in the cytoplasm (soluble forms) and plant cell wall (covalent). It has *cis* and *trans* isomers, and the *trans* form is the main isomer in native compounds (Rice‐Evans et al., 1996). The antioxidant properties of FA were compared to vitamin E, and its ability to eliminate the ROS, RNS, and free radicals has been well documented (Koh, 2012; Rice‐Evans et al., 1996; Urbaniak et al., 2013; Vieira et al., 1998). Previous reports revealed that FA not only neutralizes the free radicals but also inhibits the formation of ROS during exposure to oxidants (such as H_2_O_2_), and damages to cell or tissue conditions (Bian et al., 2015; Ogiwara et al., 2002; T. Xu et al., 2021). The ability of FA to upregulate haem‐oxygenase expression and protect cells against OT was reported (Changxing et al., 2018; Dhama et al., 2018). Moreover, FA modulates haem‐oxygenase activity by regulating the expression and translocation of transcription factor NFE2 and related factor (Nrf2) in the nucleus. With regard to this, the promoter region of the HO‐1 gene was stimulated by the antioxidant responsive element in the nucleus (Arain et al., 2018; Ma et al., 2011). In addition, beneficial roles of FA on the enzymatic antioxidants (SOD and catalase activity) in cardiomyocytes and pancreas cells of diabetic rats have been indicated (Roy et al., 2013; X. Xu et al., 2012). The current study showed that the 10 mM *t*‐FA‐treated group had statistically greater amounts of TAC compared to the negative control at 72 h, which is consistent with the previous experiments (Koh, 2012; Rice‐Evans et al., 1996; Shu et al., 2021; Urbaniak et al., 2013; Vieira et al., 1998). However, amounts of SOD were not improved by *t*‐FA treatment. Furthermore, *t*‐FA treatment did not decrease MDA, TNN, and TLHPO amounts relative to the control groups, which is in contrast to the previous study (Shayan‐Nasr et al., 2021). It seems that the differences in methodology, types of examined cells (spermatozoa vs. somatic), temperature of preservation (cold vs. incubation at 37°C), presence or absence of exogenous oxidant, and conduction at in vitro versus in vivo would affect the final response following administration.

The current experiment revealed that the addition of 25 mM *t*‐FA (dissolved in DMSO) to the ram semen extender resulted in a decrease in kinematics, viability, membrane functionality, TAC, and an increase in MDA levels of treated samples. It has been documented that high amounts of antioxidative substances disturb redox balances and act as a pro‐oxidant with the ability to increase pro‐inflammatory mediators of free radicals, stimulation of OT, and nitrosylation of proteins (Du et al., 2017; Dutta et al., 2022; Eslami et al., 2016). Therefore, the adverse effect of the drastic amounts of *t*‐FA on the different parameters of ram semen may be related to its non‐physiologic concentration for this species upon cold preservation.

The current study indicated the paradoxical effects of varying doses of exogenously added *t*‐FA on the ram semen parameters during child preservation. According to the results, 5 and 10 mM *t*‐FA improved kinematics of ram spermatozoa compared to other examined doses. However, due to mild improvement in kinematics parameters, it was not recommended as the preferred exogenous protectant for cold storage of ram semen.

## AUTHOR CONTRIBUTIONS

Ali Salimi collected the samples, worked at the laboratory, and was involved in the evaluation and semen analysis; Mohsen Eslami designed the experiment, was involved in the evaluation and analysis, and wrote and revised the manuscript; Farhad Farrokhi‐Ardabili supervised the study and edited the manuscript. All authors have read and agreed to the published version of the manuscript.

## CONFLICT OF INTEREST STATEMENT

The authors declare no conflict of interest.

### ETHICS STATEMENT

The process of semen sampling from the rams was verified by the care committee of Urmia University (IR‐UU‐AEC‐3/TDT//6160).

### PEER REVIEW

The peer review history for this article is available at https://publons.com/publon/10.1002/vms3.1117.

## Data Availability

The data that support the findings of this study are available from the corresponding author upon reasonable request.
